# Digital Health in Diabetes Care: A Narrative Review from Monitoring to the Management of Systemic and Neurologic Complications

**DOI:** 10.3390/jcm14124240

**Published:** 2025-06-14

**Authors:** Elisabetta Maida, Paola Caruso, Simona Bonavita, Gianmarco Abbadessa, Giuseppina Miele, Miriam Longo, Lorenzo Scappaticcio, Eleonora Ruocco, Francesca Trojsi, Katherine Esposito, Luigi Lavorgna, Maria Ida Maiorino

**Affiliations:** 1 Department of Advanced Medical and Surgical Sciences, University of Campania “Luigi Vanvitelli”, 80138 Naples, Italy; elisabetta.maida@studenti.unicampania.it (E.M.); simona.bonavita@unicampania.it (S.B.); gianmarco.abbadessa@unicampania.it (G.A.); giuseppina.miele@unicampania.it (G.M.); miriam.longo@unicampania.it (M.L.); lorenzo.scappaticcio@unicampania.it (L.S.); francesca.trojsi@unicampania.it (F.T.); 2 Pediatric Neurology, Santobono-Pausilipon Childrens Hospital, 80129 Naples, Italy; 3 Department of Advanced Medical and Surgical Sciences, Division of Endocrinology and Metabolic Diseases, University of Campania “Luigi Vanvitelli”, 80138 Naples, Italy; paola.caruso@unicampania.it (P.C.); katherine.esposito@unicampania.it (K.E.); mariaida.maiorino@unicampania.it (M.I.M.); 4 Department of Brain Sciences, Imperial College London, London W120BZ, UK; 5 Dermatology Unit, Department of Mental and Physical Health and Preventive Medicine, University of Campania “Luigi Vanvitelli”, 80138 Naples, Italy; eleonora.ruocco@unicampania.it

**Keywords:** diabetes, digital health, neurological complications, technology

## Abstract

**Background/Objectives**: Despite the recent advances in glucose-lowering therapy, achieving diabetes control remains challenging. With the advancing progress of innovative digital health technologies, management of diabetes is taking advantage from telehealth and telemedicine, which allow for remote assistance, virtual visits, and monitoring of diabetes-related parameters, and facilitate the exchange of documents and reports to support clinical decisions. We aim to provide an overview of the impact of telehealth and digital technologies on the care of people with diabetes, from therapeutic management to the assessment of complications. **Methods**: A comprehensive literature search was conducted using PubMed to assess the impact of digital technologies and telemedicine on diabetes care. **Results**: From the comprehensive PubMed search, 86 peer-reviewed studies were selected based on relevance, clinical significance, and methodological quality. The selected literature addressed digital health tools such as continuous glucose monitoring, connected insulin pens, automatic insulin delivery systems, mobile applications, and telemedicine systems. These interventions were associated with improved glycemic control (e.g., reduced HbA1c, increased time in range), better adherence to therapy, enhanced patient engagement, and more efficient management of complications such as neuropathy, retinopathy, and cardiovascular risk. **Conclusions**: Telehealth may offer a fully patient-centered approach to disease management through a tailored individual management plan. This may lead to an improvement in adherence to proper therapy and lifestyle, resulting in a subsequent increase in the quality of life.

## 1. Introduction

Together with cardiovascular diseases, cancer, and respiratory chronic diseases, diabetes mellitus (DM) is included in the category of “non communicable chronic diseases” (NCDs). NCDs are responsible for the highest burden of morbidity and mortality worldwide, and these four groups of diseases account for over 80% of all NCD-related premature deaths [1]. With a worldwide prevalence on the rise, type 1 diabetes (T1DM) and type 2 diabetes (T2DM) represent significant global health threats [2]. Projections show that by 2045, one in eight adults (approximately 783 million) will be living with DM, indicating a 46% increase in its prevalence rate. This scenario implies an ever-growing number of people requiring lifelong care and clinical assistance, with an increasing burden on National Healthcare Systems.

As a “pervasive” disease, diabetes requires continuous monitoring, frequent follow-ups and multidisciplinary management to optimize the achievement of therapeutic targets within a holistic person-centered approach [3]. Additionally, the management of DM has to address not only glucose-lowering therapy but also the control of the other cardiovascular risk factors and related comorbidities [3].

**Figure 1:**
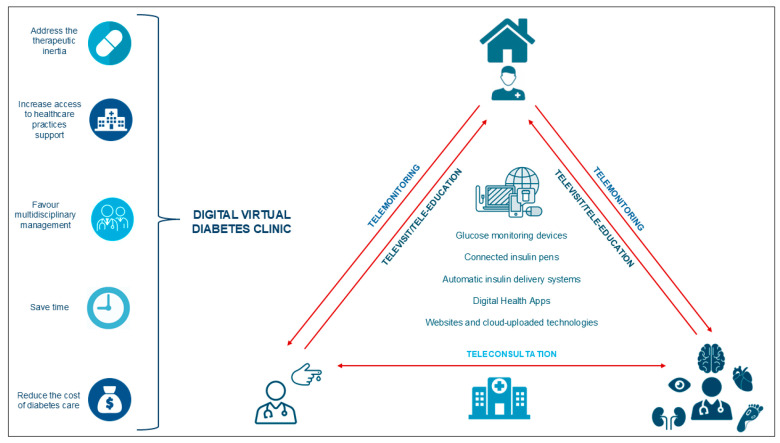
Conceptual framework of a digital virtual diabetes clinic. The figure illustrates the conceptual framework of a digital, virtual diabetes clinic, highlighting the integration of innovative digital health technologies for effective and efficient diabetes management. Through at-home blood glucose monitoring, telemedicine consultations, and personalized digital interventions, the digital virtual clinic offers a comprehensive approach to diabetes management, enhancing patient engagement and clinical outcomes.

Despite the recent advances in glucose-lowering therapy, achieving diabetes control remains challenging. Key contributors to therapeutic failure include poor patients’ adherence to medications and clinical inertia, which is the reluctance of clinicians to initiate or intensify therapy according to evidence-based guidelines [4]. To overcome the multiple and challenging obstacles to the optimal diabetes control, an organized and structured model of chronic care should be proposed for people with diabetes (PwD) [5]. With the increasing progress of innovative digital health technologies, the development of a “digital virtual diabetes clinic” has provided a valuable opportunity to implement effective and efficient diabetes management (Figure 1) [6].

The aim of this narrative review was to evaluate the impact of telehealth and digital technologies on the care of PwD, from therapeutic management to the assessment of complications. The goal was to provide a comprehensive understanding of how telehealth can revolutionize diabetes care, enhance patient care, and contribute to the efficiency of national healthcare systems.

## 2. Materials and Methods

This narrative review aimed to explore the role of telemedicine and digital health technologies in the management of diabetes, with a particular focus on their application in addressing both systemic and neurological complications. To this end, a comprehensive literature search was conducted in PubMed, targeting publications from January 2015 to December 2024; however, no lower time limit was strictly applied, and earlier studies were included if deemed conceptually or clinically relevant.

The search strategy included combinations of the following terms and keywords: “diabetes”, “type 1 diabetes”, “type 2 diabetes”, “diabetes care”, “diabetes complications”, “diabetes risk factors”, “digital health”, “digital therapeutics”, “telemedicine”, “telehealth”, “televisits”, “mobile applications”, “web-based interventions”, “continuous glucose monitoring”, “connected insulin pens”, “automatic insulin delivery”, “unmet needs”, “treatment gaps”, “patient needs”.

Inclusion criteria were as follows:

1. Peer-reviewed articles published in English;
2. Studies addressing the use of digital health tools in diabetes care, including type 1 diabetes, type 2 diabetes, and gestational diabetes;
3. Articles focusing on therapeutic strategies, monitoring technologies, and the management or prevention of complications.

We prioritized peer-reviewed studies reporting clinical outcomes in people with diabetes, including reviews, meta-analyses, randomized controlled trials, and observational studies, if relevant to the scope. Non-English publications, editorials, and opinion pieces were excluded. Relevant articles were identified and located to examine citing and cited-by articles. In total, 86 articles were selected for inclusion based on their thematic relevance and scientific contribution. The review followed the Scale for the Assessment of Narrative Review Articles (SANRA) criteria.

A narrative synthesis was developed to describe the various digital health technologies and to assess their role in symptom management, therapeutic intervention, and complication prevention in diabetes care. An overview of the principal digital health interventions, their key proximal outcomes, and the long-term complications they target is provided in Table S1 of Supplementary Materials.

## 3. Results

### 3.1. Unmet Needs in PwD

Several studies have attempted to investigate the unmet needs of people with both T1DM and T2DM [7,8,9], defined as the range of difficulties and challenges experienced by individuals throughout the course of the disease, from diagnosis to long-term therapeutic, clinical, and psychosocial management.

The DAWN2 study [8], which took its name from its predecessor, the DAWN study [7], was conducted in 17 states on more than 16,000 individuals, including patients with both T1DM and T2DM, caregivers, family members, and healthcare professionals. An online questionnaire was administered, covering several topics, such as the impact of the disease on their quality of life, diabetes management and control, support from family members, society and healthcare system, and unmet needs and hopes for the future. The results showed a clear need for psychosocial support, with 44.6% reporting distress related to the diagnosis and 13.8% showing signs of potential depression. The majority of PwD reported to be poorly involved by their physician and emphasized the need to be provided with a better education on self-management as well as psychological and social support. The issue of support was also raised by family members and caregivers who perceived diabetes as a physical and emotional burden (35.3%), with a struggle to find the appropriate way to help their loved ones.

Raposo and colleagues [9] administered an online questionnaire to 300 individuals newly diagnosed with T2DM and currently treated with glucose-lowering drugs. The responses revealed a low-to-medium level of knowledge of diabetes and related conditions prior to diagnosis (mean: 4.4 on a scale of 0 to 10); after the first visit, most responders were very satisfied with the information received. The most important unmet needs that emerged were the need to improve both the awareness and information about T2DM to enable early diagnosis; the desire for better support concerning diet and dietary habits; and lastly, the expectation for a new method of blood glucose monitoring that could be real-time and painless.

In the light of these considerations, it became essential to highlight possible solutions and assess the corresponding patient satisfaction. Digital health applied to the complexities of chronic conditions has the enormous potential to improve clinical care, thus leading to a decrease in the rate of acute and chronic complications and to an improvement of the quality of life, relieving the economic and social burden on the healthcare system and caregivers [10].

### 3.2. Telehealth and Its Application in Diabetes Care

Telehealth is defined as the utilization of digital technologies to deliver medical care, health education, and public health services by connecting multiple users in separate locations [11], indicating a model of care delivery rather than a specific treatment or therapeutic strategy. As a subset of telehealth, telemedicine refers to the exchange of medical information from one site to another via electronic communications to improve a patient’s clinical health status [11] (Table 1). As a chronic disease requiring frequent interaction between patients and healthcare providers (HCPs), diabetes could benefit from a remote approach based on digital technologies.

Numerous guidelines or international panels have identified three key areas of application for telemedicine in diabetes care to improve patients’ outcomes, increase access to care and reduce costs: 1. secondary prevention; 2. diagnosis; and 3. treatment [6,12]. In general, telemedicine allows for remote assistance and virtual visits monitoring diabetes-related parameters and facilitates communications between HCPs for consultations and the exchange of documents and reports to support clinical decisions. For these purposes, various technologies can be employed to support telehealth delivery of programs and services, including digital technologies such as continuous glucose monitoring (CGM) and automatic insulin delivery (AID), smartphone apps for mobile phones, websites or cloud-upload technologies for data download and remote monitoring algorithms, along with combinations of multiple technologies.

In a recent systematic review, Eberle and colleagues [13] addressed the role of telemedicine for T1DM and T2DM management, categorizing the available intervention protocols into four groups: real-time audio intervention, real-time video intervention, asynchronous intervention, and lastly, combined intervention. The results showed an equivalence of these approaches for the remote management of diabetic patients; however, it is imperative to adapt each of them to the specific needs of each patient [14].

A recent meta-analysis conducted by Tchero and colleagues [15] reviewed 42 randomized controlled trials (RCTs) with the aim of comparing clinical effectiveness of telemedicine in the management of diabetes with the traditional approach. The results showed that telemedicine could be more effective than traditional practice. In particular, telemedicine interventions lasting longer than 6 months tended to provide better results than those of shorter durations. Moreover, telemedicine programs have proved to be more successful in older people and T2DM, as these subjects may undergo cognitive and memory decline. Although this finding may seem a contradiction, it may be explained by the observation that in patients with mild cognitive impairment, telemedicine can compensate for possible forgetfulness in diabetes management. On the other hand, in patients with a more advanced cognitive deterioration, remote telemanagement can help caregivers in their difficult task of daily organization.

Finally, a recent review [16] sought to shed light on the cost–benefit of telemedicine for diabetes to facilitate its widespread adoption. Telemonitoring interventions together with teleophthalmology interventions for the assessment of microvascular complications in the retina were evaluated. The most important result in terms of cost savings was in teleophthalmology, which was found to be promising for early detection of microvascular ocular complications. A similar significant result was not observed for telemonitoring tools; however, several studies [17,18] showed the need to implement telemedicine techniques especially for rural populations.

#### 3.2.1. Continuous Glucose Monitoring

Glucose monitoring plays a key role in both the treatment and management of insulin-treated PwD [19,20]. A CGM system measures blood glucose levels on a near-continuous basis, providing information of blood glucose response to insulin dosing, meals, exercise, daily activities and other factors. This information enables HCPs to make necessary adjustments to the therapy as needed. Currently, two types of CGM devices are available: 1. real-time CGM (rtCGM), which continuously assesses glucose levels and are equipped with automated alarms to alerts at specific glucose thresholds and 2. intermittently scanned CGM (isCGM), which displays glucose values when swiped by a reader or a smart phone, revealing the current glucose levels. Finally, the so called “blinded” CGM measures glucose levels that are not immediately shown to the user but retrospectively analyzed by both patients and physicians to evaluate glycemic patterns and trends.

Data derived from CGM can be summarized and analyzed with software that generates reports (glucose indices, trends and statistics related to sensor usage, the time spent in the range of normoglycemia, hypoglycemia or hyperglycemia). Numerous international consensus statements recommend the Ambulatory Glucose Profile as the default report to summarize CGM data over multiple days of wear [21].

In numerous RCTs, rtCGM has demonstrated an improvement of glucose control in terms of reduction in HbA1c, increase in time in range (TIR) and a decrease in the time spent in hypoglycemia and hyperglycemia. The benefits were more significant in PwD who employed the sensor for an extended period [22,23,24,25,26,27,28,29,30,31]. Most of the studies were conducted on adults and youth with T1DM treated with continuous subcutaneous insulin infusion (CSII) or multiple daily injections of insulin (MDI). The results showed substantial benefits associated with the use of rtCGM in adults of all ages, as long as participants in the studies continued to use the device [32]. Few studies have evaluated the effect of rtCGM compared with usual care in people with T2DM [27,33] or in pregnant women with T1DM on intensive insulin regimen [34], with encouraging results.

Studies focusing on isCGM are more limited. However, there is evidence that isCGM with optional alarms is associated with a reduction in HbA1c in people with T1DM [35] and a decreased rate of hypoglycemia in T2DM [36,37]. Emerging evidence associates the use of isCGM with the reduction in diabetes-related events and all-cause hospitalizations in PwD following therapies with less intensive insulin regimen or glucose-lowering oral drugs [38,39]. Moreover, a meta-analysis of RCTs reported that, in T1DM and T2DM, both rtCGM and isCGM are associated with a modest reduction in HbA1c (−0.2%) with an overall increase in TIR of 70 min and fewer episodes of hypoglycemia and hyperglycemia [40].

Finally, T1DM, T2DM, pregnancy during intensive insulin therapy, monitoring during regular physical exercise, new onset of diabetes, and frailty have been recognized as the clinical scenarios best fitting the need for using rtCGM or isCGM, regardless of baseline HbA1c or individualized HbA1c target [41].

#### 3.2.2. Connected Insulin Pens

“Connected” insulin pens are devices capable of automatically transmitting data via Bluetooth to management apps on smartphones, representing a new valuable frontier for MDI therapy. These pens include tracking insulin pens (TIPs) and smart insulin pens (SIPs). TIPs record the last dose of insulin administered by the device and offer reminders for insulin doses through cloud connectivity. SIPs integrate data such as glucose and carbohydrates from other technological devices or apps, elaborate reports that can be shared with HCPs and provide clinical decision support through an automated insulin dose calculator based on individualized insulin therapy settings.

Emerging evidence suggests that the use of connected insulin pens is associated with increased TIR and fewer missed boluses in T1DM treated with a basal-bolus insulin regimen [42], indicating a beneficial impact on glucose control and dosing behavior [43].

In a cost-effectiveness analysis, the use of SIPs was associated with a per-patient improvement of 0.90 years in mean life expectancy and an increased 1.15 quality-adjusted life-years, along with mean cost savings over standard care [44]. These results were mainly driven by a lower frequency and delayed onset of complications. Moreover, the lower number of severe hypoglycemia events observed when these devices are used in combination with CGM may further contribute to the potential beneficial economic impact of SIPs [45].

#### 3.2.3. Automatic Insulin Delivery

AID systems include technologies that integrate data from CGM, insulin pumps and control algorithms to automatically regulate subcutaneous insulin delivery. The earlier AID system included insulin pumps with automated suspension of insulin delivery at low glucose levels (low glucose suspend, LGS) or for predictive hypoglycemia (predictive low glucose suspend, PLGS). These technologies initially enabled the glucose-responsive regulation of insulin delivery to address hypoglycemia [46,47].

The most recent closed-loop technology currently available is the advanced hybrid closed-loop system, which automatically and continually modulate basal insulin delivery in response to sensor glucose levels. It can also deliver automatic correction boluses in case of glycemic spikes but still requires the manual entry of carbohydrates consumed to calculate prandial doses. The “full” AID will automatically adjust all the required insulin, including prandial insulin at meals.

Studies focusing on various AID systems with different algorithms, pumps, and sensors have been conducted in both adults and children with diabetes [48,49,50,51]. Meta-analysis of RCTs provides evidence that AID systems may improve TIR, reduce hyperglycemia and hypoglycemia and modestly decrease HbA1c levels, compared with control therapy (conventional pump therapy or sensor-augmented pump therapy) [52,53]. PwD preference and the selection of individuals (and caregivers) capable of safely and effectively using the devices are relevant aspects for a valuable and effective therapy with AIDs [20,54]. This scenario is currently expanding with the advent of “Do-it-yourself” AID systems, which use commercially available CGM systems and insulin pumps combined with an open-source algorithm [54].

#### 3.2.4. Digital Health Apps

The recent and rapid progress in the field of diabetes technologies has led to the development of numerous mobile apps intended to increase the self-management abilities of PwD, facilitate communication between PwD and HCPs and improve compliance with tailored treatment. These Apps may help PwD improve health outcomes by supporting healthy behaviors, promoting glucose monitoring, assisting with result interpretation, guiding medication change, providing alerts for blood glucose variation, and ultimately, reducing complications.

The most commonly used digital health apps in diabetes care can be divided into three categories: 1. Apps used for tracking health and wellness, 2. apps acting as stand-alone medical devices, and 3. apps that display, download, share and/or use data from devices involved in self-blood glucose monitoring, CGM, insulin pumps or integrated system of insulin delivery and CGM, such as AID or artificial pancreas.

Apps used for tracking wellness include both nutrition and physical activity apps. These tools enable users to check the energy content of food, serve as nutrition tracker or food diary, support in planning meals and insulin dosing [55], monitor daily physical activity, count calories and set goals for exercise and weight management [56].

Glucose monitoring apps offer the possibility to aggregate data often derived from an external device (i.e., a glucometer or a CGM) and build graphs to show glucose trends. These apps are frequently integrated with a “bolus calculator”, which allows PwD to establish the insulin dose in response to the carbohydrate content of each meal, the basal insulin dose or correction doses needed in case of sudden glucose spikes. Apps supporting insulin delivery are connected to pens or insulin pumps to collect and display data, suggest boluses, download insulin and glucose data, providing a comprehensive overview for decision assistance. Finally, AID systems consist of a CGM, an insulin pump and an algorithm that adjust the basal insulin rate or provide automatic correction boluses (advanced closed loop) based on glucose levels captured by the sensor on patients [54].

Several clinical trials focusing on interventions based on apps to manage diabetes have been conducted in various population, including in children and adolescents with T1DM [57,58], adults with both T1DM and T2DM [59,60,61,62] and women with pregnancy diabetes [63].

Most studies identified the reduction in HbA1c as the primary outcome associated with the use of apps, compared with control strategies [59,60,61,62,64]. In many cases, the improvement of glycemic outcomes was enhanced with healthcare professional feedback [60,62,64] highlighting the importance of creating connections and sharing data between PwD and HCPs for an effective care process. The main limitation of these studies were the short duration of the intervention and the relatively small number of participants. Moreover, there is evidence suggesting that mobile app intervention may have beneficial impacts on medication adherence and glycemic parameters in people with T2DM [65].

However, since mobile apps designed for health and wellness are largely unregulated, there is need for regulation, standardization, and quality control, to promote a safe and effective approach, increase patient engagement, and improve disease outcomes [66].

#### 3.2.5. Websites and Cloud-Upload Technologies

Websites and cloud-upload technologies represent a relatively recent opportunity for managing diabetes remotely. This is further emphasized by the growing adoption of blood glucose monitoring systems (self-blood glucose monitoring (SBGM), isCGM, rtCGM) in clinical practice. These systems have significantly contributed to diabetes management, regardless of the type of glucose-lowering treatment, whether MDI or CSII [40,41].

Glucose monitoring data can be collected over time and sent to the cloud to review summary statistics and recognize trends of glucose levels. Downloading these data may help HCPs provide individualized recommendations for therapy, evaluate the efficacy of glucose-lowering therapy and educate patients on interventions that can improve their clinical status. In addition, remote monitoring represents a valuable strategy to intervene promptly with appropriate and immediate feedback providing treatment modification if needed.

Tools for downloading data include software and web-based platforms that are accessible by both PwD or HCPs. Data are typically summarized in tables, charts or graphs that report patterns and trends in blood glucose levels. Additionally, information on meals, insulin doses, and physical activity can also be visualized. PwD may link their personal accounts to HCP’s professional account for data sharing and remotely reviewing the information.

Downloading and reviewing glucose data through web-based platforms have been associated with a significant reduction in HbA1c in RCTs [67,68,69]. Evidence from clinical trials also suggests that telemonitoring in people with T2DM leads to improvement in diabetes control [70,71,72], comorbidities [71,73] and a better quality of life [73,74]. Moreover, a recent meta-analysis of 30 RCTs, including 4678 PwD, reported that system practicality, user engagement, patient characteristics and disease education are the main factors influencing the effect of telemonitoring on glucose control [70].

#### 3.2.6. Televisits

A higher frequency of visits has been associated by Kaufman and colleagues with a better control of HbA1c [75]. Indeed, results of their 3-year study showed a significantly lower HbA1C value for patients with 3–4 visits per year, compared to those with only 1 or 2 visits. On this basis, it should be mandatory to improve patients’ adherence to follow-up. For patients skipping appointments for reasons like work, school or distance from DM centers, telemedicine could be a suitable tool to overcome such obstacles.

The promising results of telemedicine for diabetes management were already investigated prior to the COVID-19 pandemic. Kassar et al. [76] conducted a study on 106 inmates with diabetes who agreed to remote visits with experienced endocrinologists, amounting to a total of 264 televisits. At the end of the follow-up, there was an improvement in HbA1c levels in more than half of the patients, as well as better control of cardiovascular risk factors such as hypertension and hyperlipidemia. A pre-pandemic study conducted by Tonyushkina and colleagues [77] recruited 10 young patients with DMT1 to attend remote monitoring visits. At the end of each televisit, which took 25 to 45 min, the diabetologist informed the patients about any therapeutic and lifestyle adjustments. Although no significant changes in HbA1c values were recorded, all patients were very satisfied with the telemedicine visits and wanted to continue clinical follow-up with the same modality.

The COVID-19 pandemic made it impossible for many patients with chronic diseases to attend their regular check-ups at their referral centers. As a result, the number of remote visits increased considerably, leading consequentially to an increasing number of studies on the issue with a wider number of participants. A survey of approximately 1300 patients who performed at least one televisit for clinical therapeutic management of diabetes showed an overall satisfaction with remote visits [78].

Another study [79] conducted on patients with both T1DM and T2DM investigated patients’ satisfaction with telemedicine visits. Of 111 patients, 82% were pleased with the telehealth modality and 69% were interested in continuing in remote mode even after the emergency status had ceased.

An anonymous questionnaire [80], including questions about the use and perception of telemedicine in the management of diabetes during the COVID-19 pandemic, was administered via social media in about 40 countries. A large percentage of responders (67%) said they had consulted their doctor remotely since the pandemic began, a significant increase from the 28% reported in a similar survey in 2020; furthermore, 83% of responders considered remote appointments useful.

### 3.3. Management of Diabetes Complications

#### Risk Factors

Common cardio-metabolic risk factors in patients with diabetes are represented by hypertension and hyperlipidemia (or hypercholesterolemia) and in recent years, their co-prevalence has been increasing more and more [81]. Several interventions in telemedicine have proved to significantly improve systolic and diastolic blood pressure, triglycerides, and total cholesterol serum levels [82].

Telehealth could be used to help reach the objective of reducing high cholesterol level by 1. checking tolerability when starting treatment with anti-diabetic drugs; 2. real-time monitoring of LDL cholesterol levels to ensure that the ongoing anti-diabetic treatment and a proper diet are effective in controlling the disease; 3. providing opportunities to discuss cost concerns and treatment alternatives; and 4. setting goals for healthy lifestyle changes [83].

A RCT showed that telemedicine, through home blood pressure monitoring and remote consultations, can improve blood pressure control and treatment adherence by facilitating timely therapeutic adjustments and reducing logistical barriers to in-person care [84]. By allowing the exchange of clinical data in either a synchronous or asynchronous way and by enabling remote visits, telemedicine enabled clinicians to make treatment changes at a distance, thus saving the time required to travel to the hospital and the waiting time prior to the visit [84].

Salisbury and colleagues [85] have proved that people at high risk of cardiovascular diseases who received telehealth monitoring combined with in-office visits have slightly improved their adherence to diet, exercise and medication usage and have better satisfaction with medical care when compared with people using in-person follow up alone.

A recent cross-sectional study [83] of 375 newly diagnosed T2DM patients has shown that telemedicine, in addition to standard antidiabetic therapy, helped reduce LDL cholesterol levels (110 mg/dL vs. 93.1 mg/dL, *p* < 0.001). Notably, the intervention group relied on messaging software through which they were able to keep in touch with a diabetologist and send him/her daily photos of meals and glucose values. Likewise, the clinician used this application to contact patients to make therapeutic changes or provide advice on diet and lifestyle.

### 3.4. Reduction in Diabetic Complications

#### 3.4.1. Stroke and Cardiovascular Diseases

A meta-analysis conducted by Lieber and colleagues [86] evaluated five clinical trials to assess the role of telemedicine in reducing HbA1c levels in post-stroke PwD. Compliance with diabetes treatment regimens is associated with an improvement in HbA1c blood levels and therefore, a reduction in mortality and morbidity. The enrolled patients had a digital glucometer transmitting data to the clinic at different frequencies. Four out of the five trials showed a greater reduction in HbA1c in the intervention group using telemedicine than in the traditional control group. Furthermore, in the group receiving remote consultations, there was a greater propensity to adjust treatment to achieve better glycemic control [86].

Moreover, another meta-analysis of 55 RCTs including people with T1DM (15 RTCs), T2DM (31 RTCs) or both types of diabetes (9 RCTs), revealed that when patients use telemedicine programs, better glucose monitoring was reached, with a substantial reduction in HbA1c levels compared to patients not using telemedicine programs [82]. A subgroup analysis according to diabetes type showed that T2DM patients had a significantly greater reduction in HbA1c than those with type 1 diabetes [82].

#### 3.4.2. Diabetic Neuropathy and Diabetic Foot Syndrome

Telehealth may allow self-monitoring of foot health status for preventative, diagnostic and therapeutic purposes. Different approaches are available such as audio-video consultation, photographic imaging, dermal thermography and hyperspectral imaging.

Two RCTs [87,88] compared the effect of phone/online platform tools on ulcer healing, amputation and death with standard care. In detail, Rassmussen and colleagues [87] evaluated the effectiveness of audio-video consultation in addition to outpatient visits on ulcer healing compared to standard care. Smith-Strøm et al. [88] compared a weekly phone and interactive web-based ulcer record consultation in addition to outpatient visits (every 6 weeks) to standard care (outpatient visit every 2 weeks). Both studies found no differences between the two groups in amputation and healing time, highlighting the non-inferiority of telemedicine over its traditional counterpart. A secondary outcome study of the Norwegian trial [89] explored the changes in self-reported quality of life and well-being in patients with diabetic foot receiving the telemedicine intervention compared to those receiving standard care and found no differences between the two groups. Further, patients reported outcome measures were found to be stable overtime (from baseline to several assessment time-points). In line with this evidence, two non-randomized studies [90,91] comparing video consultation with in-person outpatient visit found no difference in ulcer healing between the two approaches.

Remote ulcer area measurement from photographs could allow a continuous and reliable monitoring of ulcer evolution. Two studies [92,93] revealed a strong association between remote and live assessments of ulcer area (correlation coefficient > 0.95). Further two studies [94,95] reported a lower inter-observer variability in ulcer area measurement from a photograph compared to live assessment. Hazenberg and colleagues [96] found good feasibility and perceived usability of photographic ulcer assessment. Results of their study showed a good reliability of the combination of photographic assessment and infrared thermography in the diagnosis of foot infection (sensitivity > 60% and specificity > 79%). They also found that the combined use of both techniques was more reliable than each one on its own.

Foot temperature alteration in DFS are the results of compromised thermoregulation due to both peripheral arterial disease and neuropathy. It can be estimated through different techniques: temperature sensors based on thermistor, infrared thermography and liquid–crystal thermography. Four RCTs [97,98,99,100] explored the effectiveness of infrared thermography in ulcer prevention. Patients allocated to the intervention group were instructed to measure foot temperature in six different locations per foot, daily. Patients were invited to contact the HCP in case they revealed a 2.2 °C temperature difference between the left and the right foot in the same location for at least two consecutive days. Patients in the control group received traditional monitoring that did not include temperature monitoring. Three studies out of four [97,98,99], including, respectively, 85, 225 and 173 patients found a significant reduction in ulceration or recurrent ulcer rate in the intervention group compared to the control one (respectively: *p* = 0.01; *p* = 0.038; *p* = 0.0046). The fourth study [100] did not find any significant difference between the two groups (*p* = 0.532); however, the sample size of the patients was not as large as that reported in the other studies (45 patients).

The effectiveness of liquid–crystal thermography in ulcer prevention and diagnosis was explored only in three small clinical studies [101,102,103]. No studies are available on patients suffering from diabetes, suggesting a low applicability. Therefore, available data does not allow any reliable conclusion. Only one study [104] explored the diagnostic effectiveness of a wireless temperature sensor based on a thermistor and found that using a temperature difference between the two sides of 2.2 °C as cut off, the tool correctly recognized 97% of diabetic foot ulcers. However, the authors also found a high rate of false-positives (specificity of 43%). Even in this case the available data did not allow the researchers to reach a conclusion.

Hyperspectral imaging is a technique that measures tissue oxygenation levels and generate maps of microcirculatory changes through the collection and processing of information of the near-infrared range of the electromagnetic spectrum. Four case–control studies [105,106,107,108] on ulcers before healing revealed a reduction in oxyhemoglobin level in healed ulcers compared to no change in oxyhemoglobin level in non-healed ulcers. Conversely, three case–control studies [109,110,111] explored the specificity and sensitivity of hyperspectral imaging for prediction of ulcer healing. The first two [110,111] found a sensitivity, respectively, of 93% and 80% and specificity of 86 and 74% to predict ulcers healing in 24 weeks; conversely, the third one [109] found a sensitivity of 90% and a specificity of 86% in one month.

#### 3.4.3. Mental Illness

Mental illness is considered to play a key role among the risk factors of chronic diseases; comorbid depression predicts the onset and progression of several chronic diseases. Indeed, having strong coping mechanisms such as emotional expression, acceptance of the condition and problem solving was associated with a better quality of life and lower HbA1c levels [112]. Conversely, low scores on the PROMIS, a questionnaire assessing several aspects of the quality of life, were associated with depression and less frequent blood glucose checks [113].

A systematic review [114] including seventeen RCTs evaluated the effectiveness of digital tools in mental illness management in chronic diseases such as cardiovascular diseases, chronic obstructive pulmonary disease (COPD), stroke and diabetes. The studies included in the review described different types of tele-interventions; several offered digital educational content specific to the disease along with lifestyle advice and recommendations; others included telemonitoring applications for symptoms of depression; finally, some also provided remote psychotherapy interventions. Study results revealed a moderate beneficial effect on depression outcomes. Moreover, patients suffering from both COPD and diabetes received a higher benefit from the interventions compared to those affected by cardiovascular diseases and stroke.

## 4. Conclusions

In this narrative review, we have highlighted the established yet ever-growing role of telemedicine in the management of diabetes, covering daily management of risk factors and complications and the administration of antidiabetic therapy. The presented studies revealed an overall satisfaction among PwD and their caregivers regarding remote telemedicine management. Telehealth has demonstrated the capability to provide a fully patient-centered approach to disease management through highly personalized management plans. This has led to improved adherence to appropriate therapy and lifestyle, ultimately contributing to better quality of life.

Whilst telemedicine has clearly a wide spectrum of potential benefits, it also presents challenges that must be addressed. Firstly, many elements of the traditional approach may be missing. Physical presence and emotional connection may be limited by remote visits, and a complete objective examination may not always be possible due to technological constraints. Furthermore, real-world implementation faces additional barriers, including disparities in access to digital tools, particularly in rural or underserved areas. Inadequate broadband coverage and unaffordable costs of digital tools may prevent equitable access to telemedicine, reinforcing the existing disparities known as the digital divide. Moreover, limited digital literacy, whether due to age, educational background, or sensory or cognitive impairments, can represent a significant barrier to telemedicine access. Concerns about data privacy, cybersecurity, and unclear reimbursement policies further complicate integration into standard care models. Finally, other limitations are represented by the lack of standardized protocols of intervention, the lack of regulation and reimbursement guidelines and the limited validation of the digital devices in non-English languages. Although telemedicine is already widely implemented, its future potential lies in enabling fully patient-centered care, bringing diabetes management closer to the patient’s home.

At present telemedicine represents a valuable tool for managing diabetes and its related risk factors and complications. However certain aspects of care may still benefit from in-person interaction. For now, the best approach remains a balanced integration of telemedicine and face-to-face consultations to achieve the best possible treatment and an improved quality of life.

## Figures and Tables

**Table 1:** Definitions and components of telehealth in diabetes management. The table provides definitions of components of telehealth in diabetes management. Telehealth, defined as the utilization of digital technologies to deliver medical care and healthcare services remotely, includes different modalities such as televisit, teleconsultation, tele-education, and telemonitoring. Each modality performs distinct functions in the delivery of remote healthcare, contributing to improved patient care and outcomes in diabetes management.

TELEHEALTH	
**TELEMEDICINE IN DIABETES MANAGEMENT**	**Televisit** Televisit is a healthcare act in which the doctor interacts remotely with the patient, with the prescription of drugs or treatments during the televisit. The visit must take place in real or deferred time. A healthcare professional who is close to the patient can assist the doctor.
	**Teleconsultation** Teleconsultation represents a remote consultancy activity between doctors that allows a doctor to ask the advice of one or more doctors on the indication of diagnosis and/or choice of therapy without the physical presence of the patient, on the basis of medical information related to patient care.
	**Tele-education** Tele-education refers to any educational or learning process in which the learner is geographically apart from the instructor and/or the educational resources. Tele-education can be synchronized and not-synchronized and is commonly associated with in-person lessons or professional training procedures.
	**Telemonitoring** Telemonitoring corresponds to the transmission and/or the sharing of clinical data to a healthcare provider through electronic and web-based systems. Telemonitoring can include both non-reactive data collection and delayed and/or continuous analytic and decision-making functions.

## Supplementary Materials

The following supporting information can be downloaded at: https://www.mdpi.com/article/10.3390/jcm14124240/s1, Table S1: Digital health interventions in diabetes care and associated outcomes.

## Abbreviations

The following abbreviations are used in this manuscript:

AID	Automatic Insulin Delivery
CGM	Continuous Glucose Monitoring
COPD	Chronic Obstructive Pulmonary Disease
CSII	Continuous Subcutaneous Insulin Infusion
DM	Diabetes Mellitus
HCPs	Healthcare Providers
isCGM	Intermittently Scanned Continuous Glucose Monitoring
LGS	Low Glucose Suspend
MDI	Multiple Daily Injection of Insulin
NCDs	Non-Communicable Chronic Diseases
PLGS	Predictive Low Glucose Suspend
PwD	People With Diabetes
RCTs	Randomized Controlled Trials
rtCGM	Real-Time Continuous Glucose Monitoring
SBGM	Self-Blood Glucose Monitoring
SIPs	Smart Insulin Pens
T1DM	Type 1 Diabetes Mellitus
T2DM	Type 2 Diabetes Mellitus
TIPs	Tracking Insulin Pens
TIR	Time In Range
