# Supplementary material for: Digital Health in Diabetes Care: A Narrative Review from Monitoring to the Management of Systemic and Neurologic Complications
Source: J Clin Med. 2025 Jun 14;14(12):4240. doi: 10.3390/jcm14124240 (PMC12193807; doi:10.3390/jcm14124240)
Supplement: Supplementary file 1 [file jcm-14-04240-s001.zip › jcm-3642953-supplementary.pdf]

Digital intervention	Key proximal outcomes (clinical / behavioural)	Complications / long-term endpoints addressed
Continuous Glucose Monitoring (rtCGM / isCGM)	↓ HbA1c; ↑ Time-in-Range; ↓ symptomatic & nocturnal hypoglycaemia	Fewer hospitalisations; reduced micro- & macro-vascular risk
Connected Insulin Pens	↑ TIR; ↓ missed boluses; cost savings	Delayed diabetes-related complications through better control
Automated / Hybrid Insulin Delivery (commercial & DIY)	Large ↑ TIR; ↓ hyper- & hypoglycaemia; ↓ HbA1c	Broad risk reduction via sustained glycaemic control
Mobile Apps for Self-Management	↓ HbA1c; ↑ adherence; weight management	Behaviour-linked complication prevention
Web & Cloud Upload Platforms	↓ HbA1c through remote dose adjustment	Early therapeutic optimisation
Tele-monitoring of CV Risk Factors (BP, lipids)	↓ SBP/DBP; ↓ LDL-C; ↑ medication adherence	Cardio-renal protection
Tele-visits / Video Consultations	Stable or ↓ HbA1c; high patient satisfaction	Continuity of care, equitable access
Telehealth for Foot Care & Neuropathy	Non-inferior ulcer healing; early infection alerts	Amputation prevention; improved quality of life
Digital Mental-Health Support	↓ depressive symptoms; ↑ QoL	Enhanced self-care, reduced distress

Table S1. Digital health interventions in diabetes care and associated outcomes. Synopsis of digital health interventions in diabetes care, the most common proximal outcomes reported in literature, and the long-term complications they are intended to mitigate. Representative, widely-used technologies are listed for each category to aid reader orientation.

AID, automated insulin delivery; *BP*, blood pressure; *CBT*, cognitive behavioural therapy; *CGM*, continuous glucose monitoring; *DIY*, do-it-yourself; *DBP*, diastolic blood pressure; *HbA1c*, glycated haemoglobin; *isCGM*, intermittently scanned continuous glucose monitoring; *LDL-C*, low-density lipoprotein cholesterol; *QoL*, quality of life; *rtCGM*, real-time continuous glucose monitoring; *SBP*, systolic blood pressure; *TIR*, time-in-range. *Arrows:* ↑ denotes an improvement or increase; ↓ denotes a reduction.
